# Contextual Sensing: Integrating Contextual Information with Human and Technical Geo-Sensor Information for Smart Cities

**DOI:** 10.3390/s150717013

**Published:** 2015-07-14

**Authors:** Günther Sagl, Bernd Resch, Thomas Blaschke

**Affiliations:** 1 Department of Geoinformation and Environmental Technologies, Carinthia University of Applied Sciences, Europastrasse 4, A-9524 Villach, Austria; 2 Department of Geoinformatics—Z_GIS, University of Salzburg, Schillerstrasse 30, A-5020 Salzburg, Austria; E-Mails: bernd.resch@sbg.ac.at (B.R.); thomas.blaschke@sbg.ac.at (T.B.); 3 Department of Geography–Chair of GIScience, Heidelberg University, Berliner Strasse 48, D-69120 Heidelberg, Germany; 4 Center for Geographic Analysis, Harvard University, 1737 Cambridge Street, Cambridge, MA 02138, USA

**Keywords:** sensing, sensors, urban environments, urban dynamics, human-environment interaction, quality of life, geographic information science

## Abstract

In this article we critically discuss the challenge of integrating contextual information, in particular spatiotemporal contextual information, with human and technical sensor information, which we approach from a geospatial perspective. We start by highlighting the significance of context in general and spatiotemporal context in particular and introduce a smart city model of interactions between humans, the environment, and technology, with context at the common interface. We then focus on both the intentional and the unintentional sensing capabilities of today’s technologies and discuss current technological trends that we consider have the ability to enrich human and technical geo-sensor information with contextual detail. The different types of sensors used to collect contextual information are analyzed and sorted into three groups on the basis of names considering frequently used related terms, and characteristic contextual parameters. These three groups, namely technical *in situ* sensors, technical remote sensors, and human sensors are analyzed and linked to three dimensions involved in sensing (data generation, geographic phenomena, and type of sensing). In contrast to other scientific publications, we found a large number of technologies and applications using *in situ* and mobile technical sensors within the context of smart cities, and surprisingly limited use of remote sensing approaches. In this article we further provide a critical discussion of possible impacts and influences of both technical and human sensing approaches on society, pointing out that a larger number of sensors, increased fusion of information, and the use of standardized data formats and interfaces will not necessarily result in any improvement in the quality of life of the citizens of a smart city. This article seeks to improve our understanding of technical and human geo-sensing capabilities, and to demonstrate that the use of such sensors can facilitate the integration of different types of contextual information, thus providing an additional, namely the geo-spatial perspective on the future development of smart cities.

## 1. Introduction

Cities are complex and dynamic systems that comprise a broad range of physical and environmental features, as well as social and human-related components. The broad spectrum of technologies available today allows such features to be comprehensively quantified in unprecedented detail. These include intrinsically geographic features such as current environmental conditions (weather, air quality, *etc.*), the public’s perception of urban spaces, and the public’s individual and collective behavioral responses to a range of urban functional settings including traffic infrastructures, open spaces and open places, neighborhoods and residential areas. Such settings are of considerable importance to environment-human interactions, in particular with respect to quality of life (QoL) [1].

Smart cities, which are cities that are able to operate in a sustainable, efficient and intelligent manner [2,3], require smart infrastructure with advanced sensing capabilities that extend beyond mere technical subtleties [4], thereby possibly benefitting architects and citizens of smart cities. Systems and methods for environmental monitoring (see e.g., [5,6]) and social sensing (see e.g., [7]) are well established and cover a wide range of applications (see Table 1). However, integrating contextual information, and in particular spatiotemporal contextual information, to obtain more holistic urban analysis scenarios that take into account the human component in particular, may be able to shed new light on the behavior of complex and dynamic urban systems, thereby facilitating the development of smarter cities.

In Section 1 of this article we address context from a geospatial point of view and introduce a smart city model for interactions between humans, the environment, and technology. In Section 2 we provide a succinct analysis of the terminology used in this field and identify the main dimensions of urban geo-sensing, with a focus on measuring and quantifying different types of context. In the main body of this article (Section 3), we focus on the diverse nature of context as a key factor in the development of smart cities by proposing the use of technology-enabled contextual sensing. Finally, in Section 4 we draw some critical conclusions and address future challenges facing the overall concept of smart cities with respect to human-environmental relationships in general, and to quality-of-life aspects in particular.

### 1.1. Spatiotemporal Context for Smart Cities

Context has been described as “any information (either implicit or explicit) that can be used to characterize the situation of an entity” [8]. Contexts can be very diverse and are of critical importance in a variety of non-technical research areas including the social sciences, for example in psychology, linguistics, cognitive science, human behavior, *etc*. It is, however, beyond the scope of this article to consider the entire concept of context; the interested reader is instead referred to publications by, e.g., Brézillon [9], and Clitheroe *et al*. [10]. Instead we address certain technical aspects of context. The number of context-aware technical systems has increased significantly over the last decade [11], so that context sensing, context management, and context-aware services and applications, are now ubiquitous in computing environments [11], taking into account both external and internal contexts. External (or physical) contexts are strongly associated with the physical environment and are typically measured by physical sensors; they are therefore easy to quantify and integrate into, for example, location-based services. Internal (or cognitive) considerations address context at an individual level and can enable personalized recommendations or decision support services [12]. Both internal and external contextual factors, together with today’s (mobile) sensing technologies (refer to Section 2 for more details), support contextual modelling and contextual reasoning [13], which we believe should be integral components of smart cities. Due to their high population densities and concentrated but variable functional configurations, cities typically consist of highly dynamic urban environments, in which citizens interact continuously on both physical and social levels with their surroundings. However, the geospatial and temporal contexts (e.g., residential versus business versus industrial areas, business hours versus weekends versus holidays) within which such interactions occur are largely neglected.

Spatiotemporal contexts therefore involve much more than simply the use of locations to approximate context [14,15]. Instead they make use of “focal premises” [16] that incorporate focal data from both technical sensors and human observers. The influence of spatiotemporal contexts can be illustrated by simple examples, such as the effect that changes in traffic flow can have on the air quality [17], or that sudden rainfall can have on the normal mobility patterns within a city [18,19]. Information concerning mobility patterns needs to be individualized and context-based, as has been concluded by the authors of one of the other papers in this special issue of *Sensors* [20]. The actual (geographic) phenomena of interest therefore need to be put into (spatiotemporal) context in order that they can be better understood, as well as the possible underlying (geographic) processes [21]. Furthermore, spatiotemporal contexts are typically multifaceted in both type and magnitude (e.g., adverse wetter could range from moderate rainfall to heavy thunderstorms); spatiotemporal contexts can vary in both spatial and temporal scales and hence in spatiotemporal scope and impact (e.g., rainfall could be local or regional, it could be a shower or a steady rain, thus leading to different intensities of surface runoff); spatiotemporal contexts can sometimes be very obvious or, as is often the case, less obvious and consequently completely ignored (e.g., rainfall particulate air pollution); if spatiotemporal contexts are obvious and well-known, they can encourage context-awareness, and if they are less obvious or concealed they can still exert considerable influence on the actual geographic phenomenon of interest. The implications of spatiotemporal context can be highly individual and personal, or can be collective and social. Furthermore, a particular geographic phenomenon can at one time be the actual phenomenon of interest, and at another time part of the spatiotemporal context. For instance, noise in different parts of a city at different times of the day can be measured in order to generate dynamic noise maps for spatial planning purposes, but it can also be considered to be part of the spatiotemporal context in QoL investigations. The same noise levels may also have different implications in residential areas from in industrial areas. Contexts in general and spatiotemporal contexts in particular can therefore be seen to have an underlying complexity that can be viewed from many different perspectives—metaphorically speaking, context can be seen as a chameleon. The spatiotemporal context is, however, by no means the only determinant of either collective or individual human behavior. We therefore suggest that spatiotemporal contexts should be incorporated as one of the determinants in the design of smart city concepts, for the benefit of the affected citizens.

### 1.2. Interfaces and Interactions between Humans, the Environment, and Technology

**Figure 1:**
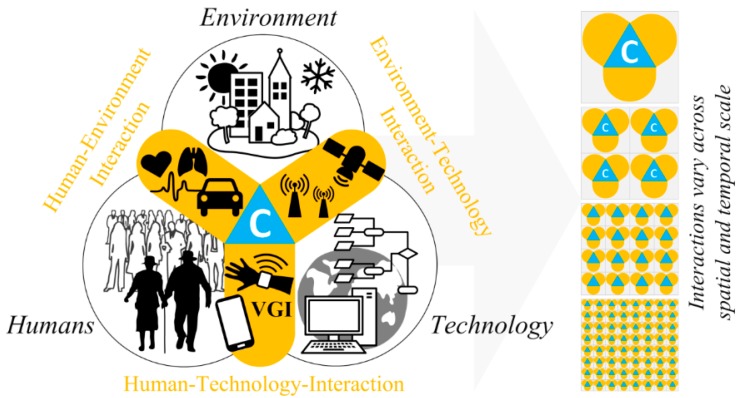
Model of smart city interactions between humans, the environment, and technology. The interfaces (in orange) between humans, the environment and technology represent the interactions between these domains, which vary across spatial and temporal scales (right side of the figure); the context (blue) is a key component at the common intersection of these interactions.

Figure 1 illustrates a conceptual geospatial perspective of three major smart city domains, namely humans, the environment, and technology, and the interactions between these domains. As mentioned previously, these domains can include (for example) current environmental conditions such as the weather, the public’s perception of urban spaces, and the public’s individual and collective behavioral responses to diverse urban settings.

The interaction model in Figure 1 highlights the previously neglected spatiotemporal component of context, which is at the heart of human-environment, environment-technology, and human-technology interactions. As indicated on the right side of Figure 1, such interactions typically vary across spatial and temporal scales, but they also vary in magnitude, scope, and impact. In other words, while the top perspective on the right hand side shows a rather generalized, aggregate or collective view of the interactions within a city, the bottom perspective shows a detailed and more individual view. The actual context within which the interactions take place can also vary within a given scale, thus influencing the vertical shifts in contextual magnitude (top-down versus bottom-up perspective in Figure 1, on the right hand side).

However, as indicated in Figure 1, only the human-technology and environment-technology interfaces are supported by sensing technologies, which are discussed in greater detail in Section 3 below. These sensing interfaces connect the real world with the digital world and allow the quantification of environment-related phenomena (labelled “Environment”) or human-related phenomena (labelled “Humans”). Direct technological sensing of human-environment interactions is, however, not possible (Figure 3), which means that these highly complex and multifaceted interactions are only very poorly represented, and only by some sort of proxy data; they are probably not represented at all in objective electronic sensor data from calibrated hardware. The capture and integration of contextual information from a geospatial point of view by leveraging sensing components, especially those from technical sensors and sensor networks, is likely to provide important support for smart city initiatives in the future.

### 1.3. Sensing Components at the Environment-Technology and Human-Technology Interfaces

We suggest that every electronic digital device can be seen as some form of sensor, but the question is, what is it sensing? The data that such digital devices and systems generate can be processed, interpreted and analyzed in a myriad of different ways. However, not all processing methods will automatically generate added-value, at least not for every situation, for every spatial and temporal scale applied, or in every instance analyzed, *etc*. This article therefore seeks to disentangle a misleading nexus of data generation and a data usage, in order to improve the QoL for urban citizens. It should be clear to any information scientist that a larger quantity of data does not automatically result in better decisions being made, or in any improvement in the QoL of citizens. However, in contextual sensing a larger quantity of data may allow contexts that have not previously been thought of, or have not previously been considered relevant, to be better understood and taken into account. This idea is implicitly based on the widely agreed concept that the processing of such data can yield useful information, and that this information may have some potential relevance, but the critical question is, to what? Nevertheless, such a supposed nexus seems to be widespread in the relatively young field of smart city research.

The number of sensors and their variety in terms of what they are able to measure within the context of smart city research has increased sharply in recent years. Progress in miniaturization and accessibility has also been accompanied by reductions in costs. A variety of sensors and sensor networks have been specifically designed to measure and quantify environmental conditions such as the weather, air quality, aerosol content, soil moisture, vegetation health, *etc*. Sensors and sensor networks now form the basis of most environmental monitoring (for example through remote sensing, *in situ* sensing, the sensor web, *etc.*).

From its original use in environmental applications, sensor technology has now spread to other fields such as human health and sporting activities, which can be seen as examples of human-environment interaction (see Figure 1). Hundreds of technologies and applications have been documented over recent years, particularly in the journal *Sensors*. We are tempted to refer to these sensors as “classic sensors” even though many of them have only been developed very recently.

Other electronic devices that were not originally designed for sensing purposes can nevertheless be leveraged for that function; examples include mobile phones, which serve as highly mobile, wireless, *in situ* components of a large-scale sensor network (the mobile phone network), which allows the whereabouts of millions of people to be monitored, whether they like it or not.

The wide spectrum of user-generated data and corresponding platforms, including Volunteered Geographic Information (VGI), Web 2.0, and social media such as Twitter, Instagram, *etc.*, mean that the multiplicity of today’s (mobile) applications used in our daily life are generating enormous amounts of data, including geo-data. Examples include recent location-based weather data, metadata, and data generated by systems in order to ensure that they are operational (“by-product” data, “overhead” data, log data, *etc.*). Weather data has been collected for longer than many other types of data. More recent examples include personal health and activity data from smart bands or other loggers, linked to smartphones. One important yet often overlooked aspect of sensing is whether or not data are being actively generated (VGI), or being reconstructed. The latter includes involuntary data from some technical process that has been triggered by a human for a specific purpose. For instance, making a mobile phone call triggers a technical process that generates the data necessary to access the mobile network and to make the phone call; such network specific relevant data are an example of involuntary data [22]. These involuntary data are typically associated with the concept of “collective sensing” [23] (see Table 1). Specifically within the context of contextual sensing for smart cities, which is the subject of this paper, it is necessary to consider the advent of this additional aspect of “involuntary sensing”, and the general question of whether or not citizens voluntarily contribute, *i.e.*, deliberately share, their data for further analysis.

## 2. Dimensions of Urban Geo-Sensing

As a basis for a holistic definition of objectively measurable and subjectively observable contextual factors and their inherent contextual information, we first established groups of available geo-sensors and frequently used terms relating to urban sensing. We then elaborated on particular characteristics of geo-sensor data in terms of the way the data is generated, the type of sensing, and the type of geographic phenomenon sensed.

Table 1 summarizes current concepts and the terminology used in urban geo-sensing under three separate headings: *in situ* technical sensors, remote technical sensors, and human sensors. These three categories are not always easily distinguishable from each other and overlap to a certain degree. While the distinction between *in situ* and remote technical sensors is quite clear (measuring in the immediate surroundings of the sensor or measuring at a distance), drawing a strict distinction between technical and human sensors can be far more challenging. Our rationale follows that of Resch [23], which defines human sensor data as human-generated measurements. These include subjective observations on the environment, social media posts, mobile phone calls and text messages, and physiological measurements by wearable body sensors. This definition implies a clear distinction between humans that generate data and humans that carry “ambient sensors” to measure external parameters (e.g., measuring air quality with a mobile sensor). Although both of these measurement types are transmitted by a digital device, the actual measurements are generated differently, in one case by humans and in the other by the sensors. This definition can entail a certain amount of overlap with the broad concept of VGI, for instance when environmental sensor readings are input into a system by a human being. In contrast, people leave behind digital traces (whether or not this is intentional) when using, e.g., the mobile network, social media platforms, credit card systems, *etc*. Such data are machine-generated by an involuntary user-induced technical process (see the mobile phone call example above) rather than generated voluntarily by a user, but nevertheless reflect the user’s whereabouts.

**Table 1:** Examples of sensors used for quantifying context to derive contextual information. Terms, related terms, characteristic context parameters, and application fields for different types of sensor.

Term	Related Terms	Characteristic Context Parameters, and Application Fields
**Technical Sensors—*in Situ* Sensors**		
Environmental sensors	Environmental monitoring, urban sensing	Meteorology and weather [24,25] Air pollution/quality monitoring [26,27,28,29,30,31] Heat island detection [28,32,33] Flood monitoring [34,35] Nuclear radiation safety [36,37,38,39]
Mobile sensors	Wearable ambient sensors, mobile sensor web	Ubiquitous measurements, e.g., through bike-mounted sensors [40,41,42,43] Disaster management [37,44,45,46,47] Embedded mobile sensor web, application-independent [48,49,50]
Pervasive sensing	Ubiquitous sensing, socially aware computing	Smart and aware environments and homes and ambient/active assisted living [51,52,53,54,55,56,57,58] Pervasive healthcare [59,60,61] RFID-based location and tracking [53,62,63] Socially aware computing [14,18,64,65]
**Technical Sensors—Remote Sensors**		
Remote sensors	Remote technical sensors and remote sensing systems, from satellite-based to terrestrial	“Classic” airborne and spaceborne optical systems [66,67,68,69,70] New developments: high resolution, hyperspectral, LiDAR, UAV [67,68,69,70,71,72,73,74] Thermal [75,76,77] Atmosphere/Aerosols [78,79,80,81]
**Human Sensors**		
People as sensors	Citizens as sensors, citizen sensing, human sensing, human sensors, humans as sensors, physiological sensors, wearable body sensors, participatory sensing, Volunteered Geographic Information (VGI)	Flood monitoring [35,82,83] Generic participatory sensing and sensing platforms (for smart cities) [84,85,86,87,88,89,90,91,92,93,94,95] Physiological parameters such as pulse, oxygen saturation, stress levels [96,97,98,99,100,101] Disaster and incident management [23,83,102] Noise mapping [103,104,105,106,107] VGI in general and in some of the above mentioned examples (including postings in social media regarding public health, air quality *etc.*) [108,109,110,111,112,113,114,115,116,117,118]
Collective sensing	Mobile phone sensing, crowd sensing, social sensing, online sensing, social media	Disaster and incident management [115,119,120,121,122] Mobility patterns and transportation [22,105,123,124,125,126,127,128,129,130] Socio-physical context estimation [97,105,131,132,133] Tourism [124,134,135] Epidemiology and disease detection [136,137,138,139]

The above table illustrates that sensor developments have opened up a whole range of new application areas—particularly through the recent proliferation of miniaturized sensors and the extensive developments in environmental sensors, coupled with the breakthrough in approaches involving human-sensors (e.g., wrist band sensors in combination with smartphones [98]). This has not only been facilitated by technological advances, but also (and perhaps to an even greater extent) by the broad adoption of sensor use in everyday urban appliances, which have produced vast quantities of sensor data that could potentially be available for urban analysis. In fact, such sensor developments, in combination with the possibility of their rapid communication, result in more anthropocentric sensing approaches that allow the public to actively contribute to the development of a smart city. In other words, current technological trends and, in particular, wearable computing [101], foster the development of “smart” citizens and their potential to capture contextual information. This means that these “smart” citizens are likely to become key contributors to the development of a smarter city than would be possible from purely technical and infrastructural contributions.

Access to these large sources of data allows measurements to be interpreted differently according to a range of contextual parameters, particularly with regard to contextual sensing. These parameters include not only data from technical sensors (which can provide a reasonably good indication of the physical context of a particular measurement), but also increasing contributions from “human sensors” (providing an indication of the emotional context of the particular person involved) in the form of physiological sensor data, social media posts, or “people as sensors” observations (see Table 1).

**Figure 2:**
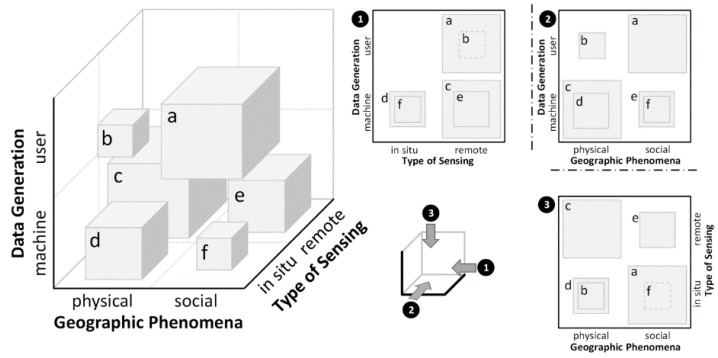
Dimensions involved in sensing (data generation, geographic phenomena, type of sensing), and some exemplary blocks (**a**–**f**) representing the amount of sensor data assigned to each dimension [140]. (**a**) VGI and mobile network traffic: associated with *in situ* sensing, social phenomena, and user-generated data; (**b**) VGI in the context of environmental status updates: associated with *in situ* sensing, physical phenomena, and user-generated data; (**c**) Satellite imagery: associated with remote sensing, physical phenomena, and machine-generated data; (**d**) Measurements from sensors and sensor networks: associated with *in situ* sensing, physical phenomena, and machine-generated data; (**e**) Human settlements extracted from satellite imagery: associated with remote sensing, social phenomena, and machine-generated data; (**f**) Numerical data at entrances to, and exits from shopping malls, public transport, *etc.*: associated with *in situ* sensing, social phenomena (e.g., mobility), and machine-generated data.

In this way urban geo-analysis approaches are able to gain a certain degree of context awareness, but it is increasingly important that they also comply with the paradigm of “socially aware computing” [64,65]. Based on the above deliberations, sensor data for context-aware analysis can be described in terms of its mode of generation, the geographic phenomena that it relates to, and the type of sensing. Figure 2 illustrates six different types of sensor data represented by six different blocks (labelled from “a” to “f”) and places them in a three-dimensional space according to the above-mentioned dimensions. Note that the sizes of the blocks shown in Figure 2 represent only a rough estimate of the proportional volume of data, for comparison purposes only.

## 3. Contextual Information as the Key for Smart Cities: A Geospatial Perspective

As discussed in Section 2, a broad mix of technologies and methods allows a variety of physical and environmental phenomena, as well as social and human-related phenomena, to be sensed and quantified. However, as we know from both published scientific literature [141,142,143,144,145,146] and our own daily experience, most such phenomena interact with each other, or at least exert some influence on each other. For instance, as described in the introduction sudden rainfall can change typical mobility patterns within a city [18,19], and changes in traffic flow can affect the air quality [17]. As discussed in Section 1.1, the geographic phenomena of interest need to be put into context in order to better understand: (i) the phenomena; and (ii) the possible underlying processes [21]. A key challenge for smart cities is therefore to take into account spatiotemporal contexts, particularly with regard to how people interact with a smart city, and how people respond to diverse urban situations.

### 3.1. Integrating Contextual Information into Geospatial Analysis for Smart Cities

As discussed in Section 1.2, human-environment interactions cannot be sensed directly using technical or human sensors; an interaction with technology is required in order to obtain a digital representation of the particular environmental or social phenomena of interest in order to explore these human-environmental interactions. Furthermore, as discussed in the Introduction, context is multifaceted and varies across both spatial and temporal scales. The overall challenge of integrating contextual information into geospatial analysis for smart cities thus involves both technical and methodological components.

The *context-aware analysis approach* [18] is an approach used to achieve context-awareness of dynamic social and environmental phenomena. In a nutshell, this approach consolidates data from various sensors and sensor networks on a common spatiotemporal basis, in order to identify and characterize potential associations and relationships between the different variables and phenomena sensed. The approach has been validated on the basis of extraordinary changes in human activity (derived from mobile network data) in the context of adverse weather conditions (derived from a set of meteorological variables).

The *context-aware analysis approach* is embedded in the *adaptive geo-monitoring framework* [21], which follows a bottom-up approach that starts from the sensing interface between the real and the digital world and then enables successive increases in the holistic understanding of geographic processes. The methodological steps involved—sensing, (context-aware) analysis, and adaptive geo-monitoring—aim to provide an improved understanding of relationships, patterns, principles, and, ultimately, processes from a geospatial perspective.

Contextual information relevant to smart cities can be considered on three scales. Contextual information on a small scale relates to individuals and their own immediate surroundings, or to very local and short-term interactions, while contextual information on an intermediate scale relates to groups of people or social communities and their close surroundings, or to interactions at a neighborhood-level and over somewhat longer periods of time; on a large scale, contextual information relates to the current population as a whole, and city-wide interactions over the long-term. Additional combinations can, however, also exist such as large-scale but short-term interactions (e.g. the overall mobility behavior in the city during the sudden onset of heavy rain). This latter combination served as an example for a case study that was previously used to validate the *context-aware analysis approach* mentioned above [18].

### 3.2. Towards a Geospatial Context-Awareness in Smart Cities

In this subsection we first focus on recent technological trends in information fusion and introduce an approach to technology-enabled contextual sensing for smart cities. We then focus on geo-sensor information fusion, specifically considering multispectral data derived from remote sensors, which can provide additional insights for smart cities, including insights into matters of public health.

#### 3.2.1. Information Fusion: From Location-Only to Human-Centered Approaches

Recent technological trends, including the increased use of the Internet of Things (IoT) and *smart home* (SH) technology, clearly indicate that the “smartness” of context-aware analyses is moving closer to the technical sensing interface between the real world and the digital world [53,94,101,147,148,149]. Such trends can lead to a new and—from a human-centered perspective—*in situ* layer of smart sensing capabilities that extend beyond location alone, and to greater context-awareness. In other words, analytical “smartness” in solving on site problems is partly “outsourced” or ”decentralized”, while still remaining embedded in the big picture of a smart city.

**Figure 3:**
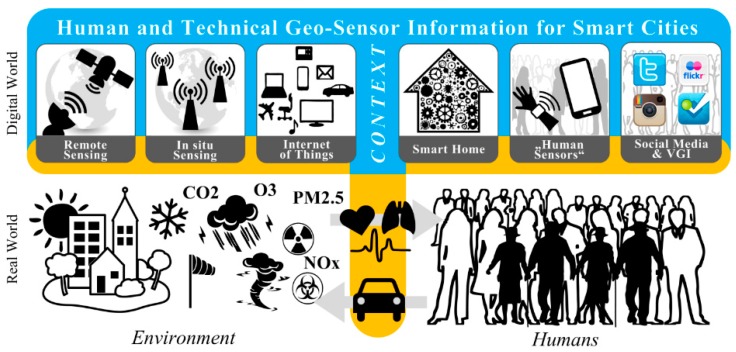
Technology-enabled contextual sensing for smart cities: context-enriched human and technical geo-sensor information for smart cities (note: interaction interfaces between the environment, humans, and technology match those in Figure 1, with emphasis placed on the sensing interface between the real world and the digital world).

The sensing interface shown in Figure 3, which is actually a combination of the environment-technology interface and the human-technology interface (Figure 1), connects the real world with the digital world. On the environmental side (Figure 3, lower left), well-established remote and *in situ* sensing technologies and methods enable real-time monitoring of physical and environment phenomena (e.g., the weather, land cover, *etc.*). On the human side (Figure 3, lower right) a variety of human sensors (people as sensors [23], citizens as sensors [108,109]) and a large body of social media and VGI data, which together can be seen as social sensors and social sensor networks [124], enable real-time “monitoring” of social and human-related phenomena (e.g., mobility, activity, *etc.*). In addition (as already mentioned above and shown in Figure 3), the IoT and the SH technology enrich the sensing interface since, as already suggested in the Introduction, “every electronic digital device can be seen as a sensor”. This enrichment takes previous sensing approaches and sensor information to the next level: integrating the “chameleon” of context by means of diverse contextual information with human and technical geo-sensor information through the use of contextual sensing.

#### 3.3.2. From Geo-Sensor Information Fusion to Smart Cities: Still a Long Way to Go

Remote sensing appears to be predestined to unravel the complexities of city landscapes However, the range of spatial and temporal sizes of urban features and the resulting range of scales and the fact that remote sensing basically provides a “bird’s-eye view” make this approach somewhat unfavorable in the context of smart cities. The number of published research papers that mention both “remote sensing” (RS) and “smart cities” is surprisingly limited, but has been increasing recently. However, the number of convincing applications of genuine uses of remote sensing for smart city applications remains limited. The authors are of the opinion that, while this may be due to a current bottleneck in smart city research, RS could eventually develop a central role in such research, in the same way as it already has in atmospheric research.

A recent example for such a development involves city-wide energy efficiency measures. Such measures require not only efficiencies in individual buildings, but also maps and balances of energy losses and potential savings [74]. Light emission and thermographic maps may therefore be used in future to provide a picture of the specific performance of individual buildings, and may consequently contribute to a better understanding of the behavior of urban citizens. A current research focus is on the contribution of cities to increased atmospheric concentrations of CO_2_, which is partly accredited to the inefficient use of energy. Another focus is on energy saving and cost reduction measures, to which smart city research can contribute by determining which areas are the least energy-efficient [70]. Remote sensing is generally limited to a “birds-eye” (nadir) view, with just a few exceptions. Unmanned Aerial Vehicles (UAVs) could be used but urban environments in most of the world’s industrialized countries impose severe restrictions on their use (for good reasons). In theory, two types of energy loss can be identified from space: (1) thermal energy loss due to the heating or cooling of buildings; and (2) excessive lighting (night-time lights), with the latter often serving as a proxy for excessive nocturnal energy use. Within the European Union, high resolution data sets such as the “Urban atlas” and the “High resolution layers”, which are available from the European Environment Agency, can be combined with other data to provide an attribution of energy emissions. The aim here is again to provide an overview, expressed for instance through heat maps and hotspot maps, that will contribute to an improved understanding of the emissions from specific areas within a city.

Another area addressed herein very briefly is public health. City authorities and urban planners need cause-effect relationship assessments between territory and health outcomes in order to create sustainable healthy urban environments, as required by ISO/DIS 37120 (see Section 4 below). For example, the number of days in which the maximum recommended concentrations of ozone, carbon monoxide, and particulate matter (PM) with a particle diameter less than 10 microns (PM10), are exceeded for more than 8 h, the number of hours exceeding maximum atmospheric concentrations of NO_2_/SO_2_, or annual concentrations of NO_2_ and PM10. Such data sets ultimately allow the influence of alternative city developments to be modeled in the context of different climate and air quality scenarios.

## 4. Conclusions and Outlook

In this paper we have attempted to integrate spatiotemporal contextual information with human and technical geo-sensor information for smart cities. We first highlighted the significance of context and introduced a model of interactions between humans, the environment, and technology within a smart city. We then elaborated on the dimensions of urban sensing and critically discussed several examples of different types of sensor used for quantifying context. A novel, human-centric contextual sensing approach for smart cities was proposed to integrate spatiotemporal contextual information at the humans-technology and environment-technology interfaces into more holistic urban analysis scenarios. We have critically examined this approach and elaborated on the benefits to citizens in terms of quality of life. The authors believe that this article will help to answer three questions on smart cities and contextual sensing.

### 4.1. Is Technology the Driving Force behind the Development of Smart Cities?

An analysis of the relevant published literature has indicated rapid increases in sensor availability, as well as improvements in sensor technology and hence sensing capabilities, particularly with respect to miniaturization and cost reduction. Smart city applications often relate to questions associated with energy consumption and mobility, and sometimes also to public health issues (mainly to do with air and water quality). The question remains as to whether or not these technologies improve the QoL for urban societies. In most of the relevant published literature (with exceptions in the fields of architecture and planning), sensor developments driven by technical requirements exceed those driven by factors relating to the QoL in general and the QoL in smart cities in particular. This may not be a favorable trend since there is at present no certainty that citizens will reap any direct benefits from the technical developments. Additionally, as discussed in Section 2 and Section 3, smart citizens could become key contributors to the development of smart cities, thereby stimulating a positive feedback loop between contributors and beneficiaries within a smart city environment.

The authors suggest that smart city developments should be guided by clearly expressed demands from the city’s inhabitants. Improving the QoL of citizens should be the overarching objective but it seems doubtful that any improvement in QoL can be demonstrated to have resulted to date from most of the developments related to the establishment of smart cities. Contextual sensing may therefore be seen as a first link in a value creation chain towards a more holistic process understanding, specifically for smart city developments. As outlined above, additional links are context-aware analysis and adaptive geo-monitoring, and also possibly geo-process mining, which can be seen as counterpart of business process mining but in the geospatial domain. We suggest that only by joining all these links together may lead to potential measures to improve urban QoL. An interesting attempt to define methodologies that can be used to steer the development of city services and produce improvements in the QoL of citizens, together with a set of indicators that can be used to measure the results, is the ISO/DIS 37120 standard (*Sustainable development and resilience of communities—Indicators for city services and quality of life*). This standard is organized into 17 “themes” but places particular emphasis on geospatial technologies including geo-sensors. Geospatial information is the key to indicator-based performance analysis in urban environments.

### 4.2. How Can Smart Cities be Identified?

The results of the literature analysis carried out for this article complement the views and viewpoints, especially the remote sensing viewpoint, expressed by Blaschke *et al*. [72]. Airborne and spaceborne remote sensing provide limited “snap-shots” of urban environments but are unable to fully capture urban dynamics. UAVs can provide supplementary information but their use is highly restricted in many countries, particularly in the more industrialized countries.

Urban areas are structurally complex 3D environments that evolve continuously with time. The problem faced is therefore how to use remote sensing technologies, in addition to vast numbers of *in situ* observations, to provide a big picture of a city. Only when this can be achieved, then the general appearance of a city, or parts of a city, can be integrated with these individual measurements into a smart city.

Furthermore, the numerous human activities that take place within urban environments are more dynamic than developments in their physical structure or changes in their functional configuration. In an attempt to achieve a better understanding of urban environments and the their inherent dynamics we have provided insights into the two currently discrete technologies of (i) remote sensing; and (ii) *in situ* sensing, and we argue that the sensor web and standardized interfaces for data and information exchange provide the opportunity to combine the strengths of both sensors and standardization, with the potential to produce new, meaningful, and useful “urban intelligence”.

### 4.3. Can Contextual Sensing Lead to a Better Quality of Life?

This answer to this question may appear obvious, but having revisited hundreds of scientific articles relevant to smart cities during the course of preparing this article, we have been surprised how techno-positivistic many of the approaches are. We conclude that it is far from clear that a larger number of sensors, more sensor applications, more complex data fusion methods, and even—as proclaimed in this article—the inclusion of context-aware approaches, will lead to any improvement in the QoL of citizens. The history of links between sensing technologies and socio-spatial applications in an urban environment is brief, and the vast majority of scientific publications relating to the development of smart cities paint a bright future. This article reveals that, despite the absence of clearly defined and widely agreed terminologies, the number of sensing technologies and sensing applications is increasing rapidly, although the distribution between sensors and applications is not even. The capabilities of *in situ* and mobile sensors are driving these developments, while the use of remote sensing technologies in smart city applications remains limited. Nevertheless, we expect the social sciences—and also the public administration and private business involved in smart city development—to require expanded urban remote sensing applications in the future. A full integration of the various sensor technologies analyzed and grouped in this article, together with strategies to capture contextual information, will help society to unravel the many critical relationships that exist between humans and their urban environments.
